# First report of *Enterobacter hormaechei* with respiratory disease in calves

**DOI:** 10.1186/s12917-019-2207-z

**Published:** 2020-01-03

**Authors:** Zhicheng Wang, Lisha Duan, Fei Liu, Yun Hu, Chaoliang Leng, Yunchao Kan, Lunguang Yao, Hongfei Shi

**Affiliations:** Henan Provincial Engineering Laboratory of Insects Bio-reactor, Henan Provincal Engineering and Technology Center of Health Products for Livestock and Poultry, China-UK-NYNU-RRes Joint Libratory of Insect Biology, Nanyang Normal University, Nanyang, People’s Republic of China

**Keywords:** *Enterobacter hormaechei*, Respiratory disease, Calf, Drug resistance

## Abstract

**Background:**

*Enterobacter hormaechei* is commonly considered a causative pathogen for nosocomial infections and it does not usually cause diseases in animals. However, researchers have recently dissociated the pathogenic *Enterobacter hormaechei* from foxes and piglets. Here, the *Enterobacter hormaechei* was first found to be associated with respiratory disease in unweaned calves in China.

**Case presentation:**

A 2-month-old calf was severely sick and diagnosed with respiratory infection by a rural veterinarian, and it died 5 days after treatment with penicillin G. The lung sample was then run through histopathological analysis and pathogen isolation. The sequence analysis and biochemical tests results showed the isolated bacterium strain to be *Enterobacter hormaechei*, and drug sensitivity tests showed resistance to all β-lactam antimicrobials and sensitivity to quinolones. Thickened alveoli septum, inflammatory cell infiltration, and erythrocyte diapedesis around the pulmonary alveoli septum were visible in lung histopathological sections. One week later, at the same farm, another calf showed similar clinical signs, and the *Enterobacter hormaechei* strain was isolated from its nasal discharge; after a week of treatment with enrofloxacin, as suggested by the results of drug sensitivity tests, this calf fully recovered.

**Conclusions:**

To the best of our knowledge, this is the first case report of calves with respiratory disease that was associated with *E. hormaechei*, and multi-drug resistance was observed in isolates.

## Background

*Enterobacter hormaechei* (*E. hormaechei*) is a species of oxidase-negative gram-negative rods that was first identified as a unique species in 1989 [1]. *E. hormaechei* is widespread in many environmental niches [2]. It is commonly considered a causative pathogen of nosocomial infections [3, 4], and it does not usually cause diseases in animals other than humans. To date, only two strains associated with disease in domestic animals have been reported: one strain of *E. hormaechei* associated with uterine infection has been identified from a dead fox [5] and another strain from the excrement of piglets with diarrhea has been isolated [6]. However, there is little evidence of *E. hormaechei* with disease in calves. In this work, we describe the first *E. hormaechei* clinical isolates from lung sample and nasal secretion in the calves. To our knowledge, this is the first isolation of this pathogen associated with respiratory disease in ruminants.

## Case presentation

Many species of bacterium, such as *Pasteurella multocida*, *Mannheimia haemolytica*, *Mycoplasma bovis*, and *Histophilus somni*, have been identified as common pathogens involved in the bovine respiratory disease complex. The aim of this report was to present pathological, microbiological, and sequence findings in a case of naturally occurring respiratory disease with *E. hormaechei* in two calves from backyard farms in the mountainous area of southern Henan Province in central China.

One 2-month-old calf (calf A) was observed to be severely sick and developed high fever (41.5~41.8 °C), depression, and reduced activity in Fangcheng City in December 2018. Further rural veterinary examination of the diseased animal showed profuse foul-smelling nasal discharge and deep abdominal respiration. It was diagnosed with bovine respiratory disease (Additional file 1). The calf was treated with penicillin G (Zhusheyong Qingmeisuna, North China Pharmaceutical Group) with 1 × 10^4^ IU per kg of b.w. in a single dose given as two doses/day for 5 days, but it died 5 days after the initiation of treatment. Then, the partial lung sample was transported to our laboratory (nearly 100 km away from the farm) for histopathological analysis and pathogen isolation by the streak plate method using a nutrient agar plate [5, 6], and after PCR detection [5, 6] and sequencing analysis of ten isolates the results showed only one species of bacterium was isolated. Further, the representive strain (HN18447) was then subjected to drug sensitivity tests and biochemical tests [5, 6]. One week later, at the same farm another 4-month-old calf (calf B) showed similar clinical signs (41.3–41.5 °C). Nasal discharge samples from this calf were also transported to our laboratory for drug sensitive testing [5, 6], and a sensitive antibiotic (enrofloxacin) (Zhusheyong Qingmeisuna, North China Pharmaceutical Group) with 2.5 mg per kg of b.w. in a single dose given as two doses/day for 5 days was chosen for the treatment of calf B. After a week of treatment, the diseased animal recovered from its respiratory infection. Sequencing analysis of ten isolates also showed only one species of bacterium was isolated, then the representive bacterial strain (HN18449) isolated from the nasal discharge was analyzed in depth as were the strains isolated from the lungs of calf A.

**Fig. 1:**
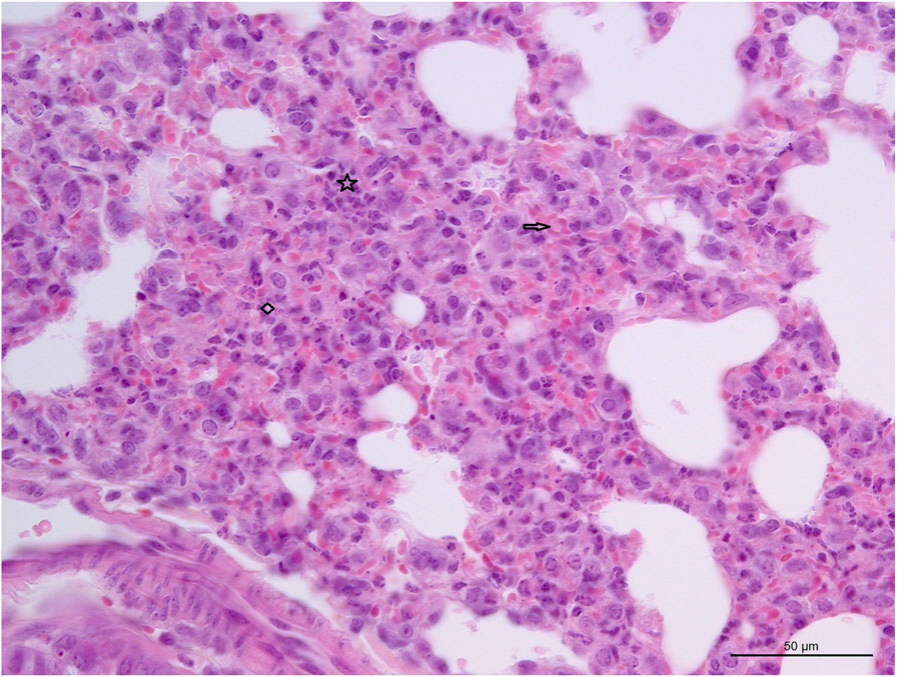
Histopathological section of the lung. Alveoli septum thickening (indicated with diamond), neutrophils infiltration (indicated with asterisk) and erythrocyte diapedesis indicated with arrow of the lung from died calf. Hematoxylin and eosin stain (Bar =50 μm)

Histopathologically, the alveoli septum in the lungs of calf A was significantly thickened, and a large number of inflammatory cells, mainly neutrophils, were infiltrated. Erythrocyte diapedesis was observable around the pulmonary alveoli septum (Fig. 1).

Lung and nasal discharge samples obtained from the two calves yielded colonies on 5% horse serum nutrient agar after 16 h of incubation at 37 °C according to culture methods in the previous research [5, 6]. The bacterial isolates were gram-negative and oxidase-negative, compatible with the general characteristics of the genus *Enterobacter*. The general primer set was used to assess the 16S rRNA gene of the all isolates [7]. Then the amplified products were recovered from the agarose gel using a gel extraction kit (Omega Bio-Tek, China), and the purified amplicons were directly sequenced in both directions using an ABI automated A373 sequencer (ABI, US). The sequence data of the 16S rRNA gene of 20 isolates from calf A and calf B indicated that all were included in the same species, and the sequences of two representive strains were deposited in GenBank under accession numbers MK774673 and MK774674. Lastly, all of the sequences were compared to the NCBI databases using a BLAST search. The BLAST results showed that the two representive strains were both *E. hormaechei*, and the 16S rDNA sequences shared 100% identity to the strains isolated from humans. The sequencing results of the 20 isolates indicated no other bacteria were isolated.

Susceptibility tests were performed using the previously described disk diffusion method and were interpreted in accordance with Clinical and Laboratory Standards Institute (CLSI) guidelines [8]. There were 26 antimicrobials that were tested. Briefly, the isolates’ inocula were plated on Mueller-Hinton agar (Beijing Solarbio Science & Technology Company, China), and the diameter of the inhibitive zone was determined following a 16-h incubation period at 37 °C in an ambient chamber. *Escherichia coli* strain ATCC 25922 was used for quality control. As shown in Table 1, the two strains isolated from calf A and calf B showed resistance to penicillin G, ampicillin, cephalexin, amoxicillin, lincomycin, roxithromycin, tetracycline, doxycycline, rifampin, azithromycin, metronidazole, clarithromycin, and vancomycin, but they were sensitive to ciprofloxacin, enrofloxacin, norfloxacin, levofloxacin, lomefloxacin, fosfomycin, nitrofurantion, florfenicol, trimethoprim sulfamethoxazole and trimethoprim.

**Table 1:** The results of drug sensitive test

Name	Sensitivity	Diameter		Name	Sensitivity	Diameter	
		HN447	HN449			HN447	HN449
Penicillin G	R	0	0	Tetracycline	R	0	0
Ampicillin	R	0	0	Doxycycline	R	0	0
Cephalexin	R	0	0	Minocycline	S/I	18	15
Amoxicillin	R	0	0	Fosfomycin	S	17	20
Ciprofloxacin	S	25	24	Nitrofurantion	S	20	22
Enrofloxacin	S	26	30	Rifampin	R	0	0
Norfloxacin	S	23	30	Azithromycin	R	0	0
Levofloxacin	S	32	28	Metronidazole	R	0	0
Lomefloxacin	S	28	30	Clarithromycin	R	0	0
Lincomycin	R	0	0	Florfenicol	S	25	25
Kanamycin	I/S	17	20	Trimethoprim sulfamethoxazole	S	24	28
Gentamicin	I	14	14	Trimethoprim	S	23	27
Roxithromycin	R	0	0	Vancomycin	R	0	0

*R* Resistant, *I* Intermediate sensitivity, *S* Sensitive, *Diameter* Diameter of inhibitive zone (mm). The zone diameter (mm) interpretive criteria for drugs: Penicillin G, Ampicillin and Amoxicillin: S ≥ 17, I:14–16, R ≤ 13; Ciprofloxacin: S ≥ 15, R ≤ 14; Enrofloxacin and Lomefloxacin: S ≥ 22, I:19–21, R ≤ 18; Norfloxacin: S ≥ 17, I:13–16, R ≤ 12; Levofloxacin: S ≥ 17, I:14–16, R ≤ 13; Lincomycin: S ≥ 13, R ≤ 12; Kanamycin: S ≥ 18, I:14–17, R ≤ 13; Gentamicin: S ≥ 15, I:13–14, R ≤ 12; Roxithromycin: S ≥ 13, R ≤ 12; Tetracycline: S ≥ 15, I:12–14, R ≤ 11; Doxycycline: S ≥ 14, I:11–13, R ≤ 10; Minocycline: S ≥ 16, I:13–15, R ≤ 12; Fosfomycin: S ≥ 16, I:13–15, R ≤ 12; Nitrofurantion: S ≥ 17, I:15–16, R ≤ 14; Rifampin: S ≥ 20, I:17–19, R ≤ 16; Azithromycin: S ≥ 21, I:18–20, R ≤ 17; Metronidazole: S ≥ 17, I:14–16, R ≤ 13; Clarithromycin: S ≥ 21, I:18–20, R ≤ 17; Florfenicol: S ≥ 22, I:19–21, R ≤ 18; Trimethoprim sulfamethoxazole: S ≥ 16, I:11–15, R ≤ 10; Trimethoprim: S ≥ 16, I:11–15, R ≤ 10; Vancomycin: S ≥ 16, I:4–8, R ≤ 2

## Discussion and conclusions

While there have been few reports of *E. hormaechei* associated with infection in animals, only a single uterine infection in a fox and intestinal infection in a piglet have been reported [5, 6]. *E. hormaechei* can be isolated from sputum, urine, exudate, wounds, blood, tissue, and organs in patients [1, 4, 9–15]. *E. hormaechei* infection is common in patients in intensive care and in infants, who often contract it through contaminated nutrition [16, 17]; these findings indicate the immunological incompetence host would be susceptible to *E. hormaechei*. In this case, two 2-month-old calves showed that respiratory disease was associated with *E. hormaechei*; this was confirmed by 16S rRNA gene sequencing and biochemical tests, and these two calves were not weaned. Considering the infection routes found in infants [11, 17], the feeding pathway might be a means by which *E. hormaechei* spreads in calves. Previous studies have reported that *E. hormaechei* can contaminate infant formula, and such cases have been found in Italy, the Czech Republic, and Holland [11, 18], so the risk of animal-to-human transmission is not negligible.

A large number of respiratory diseases have been described in calves, with pathogens including a variety of bacteria [19], but no report of calf pneumonia associated with *E. hormaechei* has been published. Compared to the signs of the calf infected by other bacterium such as *Pasteurella multocida*, non-specific clinical findings were observed in this case, and this may lead to misdiagnosis and inappropriate treatment. Currently, β-lactam antimicrobials (penicillin G, ampicillin, cephalexin, and amoxicillin) are extensively used in the treatment of calf respiratory infections in China [20]. In this case, the first calf was treated with penicillin G for 5 days, but it still died. Drug sensitivity testing showed that the *E. hormaechei* strains isolated from the lungs of calf A were completely resistant to β-lactam antimicrobials, so the failure of treatment of the first calf was not unexpected; the strains were also completely resistant to the nine other antimicrobials. Similarly, the strain isolated from the dead fox showed the same resistance to β-lactam antimicrobials and tetracycline antibiotics and the same sensitivity to quinolones (ciprofloxacin and enrofloxacin) [5]. O’Hara reported that the majority of strains isolated from humans are resistant to ampicillin, cefoxitin, and cephalothin [1], and a strain isolated from a female inpatient in Brazil was also resistant to all of the β-lactam antimicrobials tested, and it harbored two mobile genetic elements (Tn4401 and ISAba125) which play roles in the production of *Klebsiella pneumoniae* carbapenemase and New Delhi metallo-β-lactamase, respectively [12]. Whether the isolates in this case share this mechanism of resistance to β-lactam drugs remains unclear. Lately, in China, the strains isolated from patients have shown that mobile genetic elements could transmit their ability to resist β-lactam to the wild-type recipient bacterium [15], and additional investigations are warranted to address this issue.

To the best of our knowledge, this is the first case report of calves showing respiratory disease to be associated with *E. hormaechei*. The presence of this pathogen in the two calves separated by an interval of 1 week in the same farm suggests that the *E. hormaechei* might be a pathogen in calves. However, the limited number of animals (2) involved in this case is not sufficient to allow us to draw that conclusion. Broader investigations of *E. hormaechei* associated with respiratory disease are need to establish the relationship between the disease and the bacteria. A future work evaluating calves experimentally infected with the isolated strains would help establish the pathogenicity of *E. hormaechei* in the new emerging host and whether the infection routes through feeding pathway found in infants [11, 17] was utilized by *E. hormaechei* in spreading in calves could be investigated in further research.

## Supplementary information

**Additional file 1.** Calf’s clinical appearance. Abdominal respiration movements were observed.

## Data Availability

All data generated or analyzed during this study are included in this published article Sequences obtained in the present study are deposited in GenBank under accession numbers MK774673 and MK774674.

## Abbreviations

16S rRNA: 16S ribosomal RNA
*E. hormaechei*: *Enterobacter hormaechei*
